# Letter to the editor regarding to ‘cardiovascular safety of janus kinase inhibitors in inflammatory bowel disease: a systematic review and network meta-analysis’

**DOI:** 10.1080/07853890.2025.2586347

**Published:** 2025-11-10

**Authors:** Shizhen Liu, Lijuan Zhang

**Affiliations:** ^a^ Department of Anorectal Surgery, Linyi Traditional Chinese Medicine Hospital, Linyi, Shandong, China; ^b^ Department of Gastroenterology, Linyi Traditional Chinese Medicine Hospital, Linyi, Shandong, China

To the Editor

The recently published systematic review and network meta-analysis on the ‘Cardiovascular safety of Janus kinase inhibitors in inflammatory bowel disease: a systematic review and network meta-analysis’ provided evidence summarizing the cardiovascular outcomes associated with these emerging therapies [1]. Nevertheless, some issues warrant further discussion.

First, the authors’ approach appears to equate ‘absence of evidence’ with ‘evidence of absence’. While the pooled odds ratios for major adverse cardiovascular events (MACE), venous thromboembolism (VTE), and composite cardiovascular events (CVE) were not statistically significant, most trials included fewer than 500 participants and lasted less than one year. Such limited event counts lead to extremely low statistical power to detect meaningful differences. Even a moderate risk doubling could be easily masked under such sparse data conditions [2]. Have the authors performed a formal trial sequential analysis to ascertain whether the cumulative evidence has reached the required information size to exclude clinically important effect sizes?

Second, the meta-analytic framework blends distinct JAKinibs with profoundly different pharmacodynamic profiles, such as pan-JAK inhibition by tofacitinib versus TYK2-selective inhibition by deucravacitinib, into a single network. This indirect comparison assumes a class-effect equivalence that may not be biologically or pharmacologically plausible. Existing mechanistic and clinical data suggest that varying JAK selectivity yields heterogeneous impacts on lipid metabolism, interferon signalling, and thrombosis pathways [3]. The interpretation that all JAKinibs share comparable cardiovascular risk therefore risks oversimplifying a mechanistically diverse drug class.

Third, the ranking of agents by cardiovascular safety using P-scores or SUCRA values, while quantitatively elegant, may convey an illusion of precision unsupported by the underlying evidence. When event rates are exceedingly low and credible intervals span orders of magnitude, minor numerical differences in ranking carry no clinically meaningful distinction. Could the authors clarify how they accounted for the instability inherent to zero-event corrections and whether these rankings were robust under Bayesian sensitivity analyses?

Fourth, the review’s conclusions appear to disregard accumulating observational and post-marketing data outside the narrow RCT framework. Real-world data from rheumatoid arthritis and psoriasis cohorts indicate increased MACE or VTE risks with tofacitinib and baricitinib, particularly among older patients or those with preexisting cardiovascular risk factors [4,5]. While disease contexts differ, the shared pharmacological pathways urge caution. Should not the cardiovascular risk assessment of JAKinibs in IBD also triangulate evidence from pharmacovigilance and registry studies, rather than relying solely on underpowered RCTs?

In conclusion, this network meta-analysis is an important effort toward understanding JAKinib safety in IBD, yet its findings should be interpreted with restraint. Before accepting all JAK inhibitors as cardiovascularly benign, adequately powered long-term comparative and real-world studies are essential.

## Data Availability

Data sharing is not applicable to this article as no new data were created or analyzed in this study.
